# Essential oils of *Citrus aurantifolia*, *Anthemis nobile* and *Lavandula officinalis*: in vitro anthelmintic activities against *Haemonchus contortus*

**DOI:** 10.1186/s13071-018-2849-x

**Published:** 2018-04-25

**Authors:** Luis Eduardo Ferreira, Bruno Iglesias Benincasa, Ana Lúcia Fachin, Silvia Helena Taleb Contini, Suzelei Castro França, Ana Carolina Souza Chagas, Rene Oliveira Beleboni

**Affiliations:** 1 Unidade de Biotecnologia, Universidade de Ribeirão Preto (UNAERP), Ribeirão Preto, São Paulo Brazil; 2 Embrapa Pecuária Sudeste (CPPSE), São Carlos, São Paulo Brazil

**Keywords:** Botanical anthelmintics, In vitro assays, Plants, Gastrointestinal nematodes, Small ruminants

## Abstract

**Background:**

Infections of sheep with gastrointestinal parasites, especially *Haemonchus contortus*, have caused serious losses in livestock production, particularly after the emergence of resistance to conventional anthelmintics. The search for new anthelmintic agents, especially those of botanical origin, has grown substantially due to the perspective of less contamination of meat and milk, as well as other advantages related to their cost and accessibility in less developed countries. The aim of this study was to evaluate the in vitro anthelmintic activity of essential oils of the plant species *Citrus aurantifolia*, *Anthemis nobile* and *Lavandula officinalis* against the main developmental stages of the parasite *H. contortus*.

**Results:**

Plant species were selected based on substantial ethnopharmacological information. Analysis of the composition of each oil by gas chromatography coupled to mass spectrometry (GC-MS) demonstrated the presence of limonene (56.37%), isobutyl angelate (29.26%) and linalool acetate (35.97%) as the major constituents in *C. aurantifolia*, *A. nobile* and *L. officinalis*, respectively. Different concentrations of each oil were tested in vitro for their capacity to inhibit egg hatching (EHT), larval development (LDT) and adult worm motility (AWMT) using a multidrug-resistant strain of *H. contortus* (Embrapa 2010). The IC_50_ values obtained for the oils of *C. aurantifolia*, *A. nobile* and *L. officinalis* were 0.694, 0.842 and 0.316 mg/ml in the EHT and 0.044, 0.117 and 0.280 mg/ml in the LDT, respectively. The three oils were able to inhibit adult worm motility completely within the first 8–12 h of observation in the AWMT.

**Conclusions:**

The present results demonstrate significant anthelmintic activity of the three oils against the different developmental stages of *H. contortus*. Furthermore, this study is of ethnopharmacological importance by validating the anthelmintic activity of the oils studied. Although new experiments are necessary, these data contribute to the development of pharmaceutical-veterinary products for sheep farming by opening up new therapeutic possibilities against gastrointestinal infections caused by *H. contortus*.

## Background

There is a great interest in sheep farming because of the high added value of its commercial products [1]. This livestock sector has faced serious problems related to gastrointestinal infections caused mainly by the parasite *Haemonchus contortus* [2]. The epidemiological importance of this nematode species is related to its wide geographical distribution and pathogenicity. In addition, this parasite is a cause of significant economic losses for the sheep livestock industry around the world [3]. As a result of the inadequate management of infections, resistance against the main classes of commercially available antiparasitic agents (benzimidazoles, macrocyclic lactones and imidazothiazoles) has been developed by this species [4, 5], reducing their therapeutic efficacies and consequently increasing associated economic impacts [6].

A technical resource for the prevention and treatment of different veterinary diseases is the use of medicinal plants. Plants have already been used for medical purposes by ancient civilizations and are currently considered a more sustainable and more easily accessible therapeutic and/or preventive alternative to synthetic drugs [7, 8]. These potential advantages have favored the search for new herbal or plant-derived agents for veterinary use, particularly those for the control of gastrointestinal parasites, including *H. contortus* [9].

The choice of the plant species for pharmacological studies is important and should be based on solid operational strategies and relevant ethnopharmacological/chemotaxonomic data [10]. Within this context, *Citrus aurantifolia, Anthemis nobile* and *Lavandula officinalis* were traditionally used in folk medicine as anthelmintics against different etiological agents. They are widely distributed in Brazil, and are relatively good producers of essential oils. The oils extracted from the plants are a rich mixture of terpenes and terpenoids [11]. Many of these compounds have already proven their anthelmintic activities against different parasites, including *H. contortus*. Furthermore, the botanical families and genera to which *C. aurantifolia* (family Rutaceae; genus *Citrus*), *A. nobile* (family Asteraceae; genus *Anthemis*) and *L. officinalis* (family Lamiaceae; genus *Lavandula*) belong are traditionally known for their anthelmintic activity against *H. contortus* [12–15].

Despite the great potential of *C. aurantifolia*, *A. nobile* and *L. officinalis* as anthelmintics, there are no data on the potential activities of the essential oils of these plants against *H. contortus*. Therefore, the aim of the present study was to investigate the in vitro anthelmintic activity of essential oils obtained from the fruit peel of *C. aurantifolia* (Christm.) Swingle (Rutaceae); flowers of *A. nobile* (syn. *Chamaemelum nobilis* (L.) All.) (Asteraceae); and flowers of *L. officinalis* (Chaix & Kitt.) (Lamiaceae), against different developmental stages of the parasite *H. contortus*.

## Methods

### Plant material and essential oils

The essential oils of *Citrus aurantifolia*, *A. nobile* and *L. officinalis* were purchased from Kampo de Ervas Ind. & Com. Ltda-ME (Ribeirão Preto, SP, Brazil), accompanied by the technical datasheets and serial numbers of each species thus: Lot. No. 116, density (*d*) = 0.868; Lot. No. 297, density (*d*) = 0.908; Lot. No. 229, density (*d*) = 0.891, respectively. The essential oils were extracted by hydrodistillation from the fruit peel of *C. aurantifolia* and from the flowers of *A. nobile* and *L. officinalis*.

### GC-MS analysis

Samples of each essential oil were analyzed by gas chromatography coupled to mass spectrometry (GC-MS) (Varian, model Saturn 2100T) using the following technical conditions: DB-5 capillary column (30 m × 0.25 mm × 0.25 μm); gun: 240 °C; detector: 230 °C; electron impact: 70 eV; carrier gas: He; flow rate: 1.0 ml/min; split: 1/20; temperature program: 60–240 °C, 3 °C/min; injection volume: 1 μl. The chemical compounds were identified by comparison of their mass spectra with the GC-MS database (Nist 62 library) and Kovats retention index [16].

### Animals and artificial infection with *Haemonchus contortus*

Two male Santa Inês sheep (4–6 months-old, 20–30 kg) were used as feces donors for all experimental protocols. The animals were purchased in the region of Ribeirão Preto (SP, Brazil) and kept at the experimental farm of the University of Ribeirão Preto (UNAERP), receiving ration and hay two times per day and water *ad libitum*.

For artificial and monospecific infection with *H. contortus*, the animals were first treated with Zolvix® (Monepantel, Novartis Animal Health, 2.5 mg/kg body weight; Dundee, UK) according to the manufacturer's recommendations. After 14 days, a total reduction in egg load was confirmed by counting the number of eggs per gram of feces (epg = 0). The multidrug-resistant *H. contortus* strain, Embrapa 2010, was used for monospecific infection and each animal was inoculated orally with approximately 4000 third-stage (L3) larvae. After 28 days of incubation, infection was confirmed by epg count. Animals with a count of 1500 to 5000 epg were considered parasitologically competent as feces donors for the in vitro assays. According to Chagas et al. [17], strain Embrapa 2010 was isolated from naturally contaminated animals of Embrapa Pecuária Sudeste, São Carlos, SP, and is characterized as resistant to anthelmintics of the benzimidazole, macrocyclic lactone and imidazothiazole classes.

### In vitro anthelmintic assays

#### Egg recovery technique

The egg hatch test (EHT) and larval development test (LDT) described below were carried out after the recovery of *H. contortus* eggs according to the method described previously [18]. Approximately 20 g of feces were collected from the rectal ampulla of monospecifically infected animals and immediately filtered under running water (37 °C) through sieves with a mesh size of 500, 150, 90 and 20 μm, respectively. The eggs retained on the last sieve were washed, centrifuged for 5 min at 1811× *g* with distilled water, and recovered in super-saturated saline by simple flotation. After recovery, the eggs were stored on the 20 μm sieve for abundant washing with distilled water to produce a final and quantified aqueous solution of eggs.

#### Egg hatch test (EHT)

For the EHT, 24-well plates (TPP ref. no. 92024) containing aqueous solutions of approximately 100 eggs/well were used as proposed by Katiki et al. [19]. The treatments consisted of different concentrations of each essential oil (50, 25, 12.5, 6.25, 3.125, 1.562, 0.781, 0.390, 0.195 and 0.097 mg/ml) in a final volume of 0.5 ml/well completed with distilled water. The oils were solubilized in distilled water and Tween 80 (3%, v/v). After incubation for 24 h (27 °C), the number of eggs and first-stage (L1) larvae were counted under an inverted microscope and compared to the positive (thiabendazole, 0.025 mg/ml) and negative controls (3% Tween 80, v/v). The results were expressed as the mean percentage of inhibition of egg hatching in three independent experiments performed in triplicate.

#### Larval development test (LDT)

The LDT was also carried out in 24-well plates containing approximately 100 eggs/well. Following the method described previously [20], 80 μl of nutritive medium (*Escherichia coli*, yeast extract, amphotericin B) was added to each well and completed with distilled water to a final volume of 250 μl. After incubation for 24 h (27 °C), different concentrations of each essential oil (3.0, 1.5, 0.75, 0.375, 0.187, 0.0937, 0.0468, 0.0234, 0.0117, 0.0058 and 0.0029 mg/ml) were solubilized in distilled water and DMSO (1%, v/v) and added to each well, resulting in a final volume of 0.5 ml. After 6 days, the differential count of L1, L2 and L3 larvae was performed and compared to the data obtained for the positive (levamisole, 20 mg/ml) and negative controls (1% DMSO, v/v). The results are the average of three replicates and are expressed as the percentage of larval development inhibition of three independent experiments.

#### Adult worm motility test (AWMT)

The AWMT was performed after necropsy for removal of the abomasum and recovery of adult parasites from sheep infected artificially with the resistant isolate Embrapa 2010, using the method described previously [21]. In 24-well plates, five adult parasites/well were treated separately with each essential oil at final concentrations of 50, 5.0 and 0.5 mg/ml. The oils were solubilized in PBS + antibiotic solution (penicillin/streptomycin) (4%) + Tween 80 (2%) in a final volume of 1 ml/well. The plates were kept in an oven at a controlled temperature (37 °C) throughout the experiment and mobile and immobile adult parasites were counted at intervals of 2 h. The total time of the experiment was defined when extensive parasite death was observed in the negative control (24 h). In fact, the effect of essential oils on the adult worm motility was estimated considering the natural nematode motility inhibition in the negative controls. Levamisole (20 mg/ml) was used as positive control and the negative controls consisted of PBS + antibiotic solution (penicillin/streptomycin) (4%) + Tween 80 (2%) and PBS + antibiotic solution (penicillin/streptomycin) (4%). The results are the average of three replicates and are expressed as the percentage of motility of adult parasites when exposed to different concentrations of essential oils of three independent experiments.

### Statistics

One-way ANOVA followed by Tukey's test (*P* < 0.05) was used for statistical analysis. Nonlinear regression/logarithmic distributions were applied to calculate the minimum concentration that inhibited 50% (IC_50_) of egg hatching or larval development.

## Results

### GC-MS results

Analysis by GC-MS identified 91.17%, 90.54% and 91.99% of the present chemical constituents in oils of *C. aurantifolia*, *A. nobile* and *L. officinalis*, respectively. A wide variety of compounds were found in each oil; however, the major constituents were limonene (56.37%), β-pinene (11.86%) and γ-terpinene (11.42%) in *C. aurantifolia* oil; isobutyl angelate (29.26%), isoamyl angelate (15.27%) and α-thujene (8.92%) in *A. nobile* oil, and linalool acetate (35.97%), trans-sabinene hydrate (29.17%) and camphor (5.54%) in *L. officinalis* oil (Table 1).

**Table 1:** Relative percentage (%) of compounds from essential oils determined by CG-MS

RI	Component	*C. aurantifolia*	*A. nobilis*	*L. officinalis*
914	Amyl acetate	–	0.10	–
1062	Artemisia ketona	–	5.84	–
1505	β-Bisabolene	0.89	–	–
1169	Borneol	–	0.18	2.14
811	Butyl acetate	–	1.11	0.80
954	Camphene	–	0.52	0.18
1146	Camphor	–	–	5.54
1419	(E)-Caryophyllene	–	–	2.37
1031	1.8-Cineole	–	3.97	4.87
952	Cyclohexanone <3-methyl->	–	0.62	–
1024	ρ-Cymene	4.17	–	–
804	Ethyl butanoate	–	0.41	4.08
990	Furfuryl acetate	–	0.61	–
1101	Hexyl propanoate	–	0.25	–
1146	Isoamyl angelate	–	15.27	–
1009	Isoamyl isobutyrate	–	2.73	–
1160	Isoborneol	–	–	0.30
1051	Isobutyl angelate	–	29.26	–
911	Isobutyl isobutyrate	–	3.93	–
1090	Isobutyl tiglate	–	0.71	–
876	Isopentyl acetate	–	–	0.42
1290	Lavandulyl acetate	–	–	0.77
1029	Limonene	56.37	–	0.52
1257	Linalool acetate	–	–	35.97
1088	ρ-Mentha-2,4(8)-diene	–	–	0.24
988	Myrcene	0.67	–	0.16
1195	Myrtenol	–	0.77	–
1050	(E)-β-Ocimene	–	–	0.74
1037	(Z)-β-Ocimene	–	–	0.56
1132	Allo-Ocimene	–	–	0.61
991	3-Octanol	–	0.56	–
1055	Pentyl isobutanoate	–	0.53	–
979	β-Pinene	11.86	–	–
1170	Pinocampheol	–	–	0.31
1175	Cis-Pinocamphone	–	0.37	–
1164	Pinocarvone	–	3.15	–
975	Sabinene	–	6.27	0.22
1098	Trans-Sabinene hydrate	–	–	29.17
1221	Cis-Sabinene hydrate acetate	–	–	0.14
1142	Trans-Sabinol	–	4.47	–
1177	4-ol-Terpinen	–	–	0.74
1059	γ-Terpinene	11.42	–	–
1199	γ-Terpineol	–	–	0.85
930	α-Thujene	2.85	8.92	0.31
1431	Cis-Thujopsene	1.20	–	–
967	Verbenene	1.74	–	–
	Not identifield	8.83	9.46	8.01
	Total analyzed	91.17	90.54	91.99

*Abbreviation*: RI, retention index

### In vitro anthelmintic activity

The EHT results for the three essential oils showed an inhibitory effect on egg hatching higher than 85–90% for a concentration range of 3.125 to 50 mg/ml: *L. officinalis* (ANOVA: *F*_(11, 24)_ = 1394, *P* < 0.0001); *A. nobile* (ANOVA: *F*_(11, 24)_ = 571.2, *P* < 0.0001); *C. aurantifolia* (ANOVA: *F*_(11, 24)_ = 703.6, *P* < 0.0001) The IC_50_ values were 0.316 mg/ml for *L. officinalis*, 0.694 mg/ml for *C. aurantifolia*, and 0.842 mg/ml for *A. nobile*. These results are shown in Table 2. The inhibitory effect on egg hatching exhibited a dose-dependent response profile for all oils studied.

**Table 2:** Effects (mean percentage ± SE) of tested essential oils on inhibition of egg hatching of *H. contortus*

Concentration (mg/ml)	*C. aurantifolia*	*A. nobilis*	*L. officinalis*
50	100.0 ± 0.0^a,b^	99.0 ± 0.38^a,b^	100.0 ± 0.0^a,b^
25	100.0 ± 0.0^a,b^	99.4 ± 0.87^a,b^	100.0 ± 0.0^a,b^
12.5	98.9 ± 1.87^a,b^	97.0 ± 2.38^a,b^	99.8 ± 0.30^a,b^
6.25	96.4 ± 2.61^a,b^	95.7 ± 2.13^a,b^	99.4 ± 0.94^a,b^
3.125	95.9 ± 2.41^a,b^	86.4 ± 4.71^b^	92.3 ± 3.03^b^
1.562	86.9 ± 2.33^b^	74.5 ± 3.58^b^	82.8 ± 3.62^b^
0.781	56.2 ± 3.97^b^	63.3 ± 4.12^b^	85.0 ± 3.15^b^
0.390	42.1 ± 4.70^b^	26.6 ± 1.46^b^	81.8 ± 1.36^b^
0.195	14.9 ± 3.89^b^	13.4 ± 2.33^b^	9.5 ± 1.54^b^
0.097	13.0 ± 1.44^b^	14.1 ± 2.60^b^	6.8 ± 1.69
	IC_50_ = 0.694 mg/ml	IC_50_ = 0.842 mg/ml	IC_50_ = 0.316 mg/ml
	*R*^2^ = 0.985	*R*^2^ = 0.978	*R*^2^ = 0.950
Thiabendazol (0.025 mg/ml)	100.0 ± 0.0^b^	100.0 ± 0.0^b^	100.0 ± 0.0^b^
Tween 80 (3%)	7.6 ± 0.85	7.4 ± 3.94	3.5 ± 0.82

*Abbreviations*: IC_50_, inhibitory concentration 50%; *R*^2^, nonlinear correlation coefficient

^a^No statistically significant difference between positive control and treatments

^b^Significant difference between positive control or treatments with negative control (ANOVA, followed by Tukey test; *P <* 0.05)

As observed in the EHT, the anthelmintic activity of all tested oils was also promising in the LDT. The rate of inhibition of larval development by the essential oil of *C. aurantifolia* was greater than 85% at a concentration of 0.187 mg/ml (ANOVA: *F*_(11,24)_ = 2540, *P* < 0.0001) or higher, with this oil showing the lowest IC_50_ (0.044 mg/ml) among the oils tested. For the oil of *A. nobile*, the IC_50_ was 0.117 mg/ml, achieving an inhibition greater than 85% at a concentration of 0.375 mg/ml (ANOVA: *F*_(11, 24)_ = 1309, *P* < 0.0001). The results obtained for the *L. officinalis* oil showed inhibition greater than 85% only for concentrations above 1.5 mg/ml (ANOVA: *F*_(11,24)_ = 1485, *P* < 0.0001) and the IC_50_ value was the highest among the oils tested (0.280 mg/ml) (Table 3). The inhibitory effect of the oils on larval development was dose dependent.

**Table 3:** Effects (mean percentage ± SE) of different concentrations of essential oils on inhibition of larval development of *H. contortus*

Concentration (mg/ml)	*C. aurantifolia*	*A. nobilis*	*L. officinalis*
3.0	–	100.0 ± 0.0^a,b^	95.3 ± 1.84^a,b^
1.5	100.0 ± 0.0^a,b^	100.0 ± 0.0^a,b^	89.8 ± 0.88^b^
0.75	100.0 ± 0.0^a,b^	97.2 ± 2.26^a,b^	81.4 ± 1.83^b^
0.375	90.1 ± 1.68^b^	86.4 ± 2.34^b^	64.2 ± 1.79^b^
0.187	85.4 ± 2.62^b^	67.1 ± 3.26^b^	35.9 ± 0.98^b^
0.0937	71.5 ± 1.35^b^	46.9 ± 1.18^b^	11.5 ± 0.95
0.0468	61.8 ± 1.16^b^	31.3 ± 2.81^b^	12.0 ± 2.83
0.0234	41.2 ± 0.81^b^	27.4 ± 1.61^b^	9.9 ± 2.02
0.0117	14.7 ± 1.81^b^	20.5 ± 1.28^b^	8.8 ± 1.44
0.0058	10.8 ± 0.35	12.4 ± 1.17	6.3 ± 0.64
0.0029	9.1 ± 0.59	–	–
	IC_50_ = 0.044 mg/ml	IC_50_ = 0.117 mg/ml	IC_50_ = 0.280 mg/ml
	*R*^2^ = 0.983	*R*^2^ = 0.989	*R*^2^ = 0.994
Levamisol (20 mg/ml)	99.8 ± 0.21^b^	99.8 ± 0.21^b^	99.8 ± 0.21^b^
DMSO (1%)	9.3 ± 2.16	7.8 ± 0.72	7.5 ± 3.17

*Abbreviations*:C_50_, inhibitory concentration 50%; *R*^2^, nonlinear correlation

^a^No statistically significant difference between positive control and treatments

^b^Significant difference between positive control or treatments with negative control (ANOVA, followed by Tukey test; *P <* 0.05)

In the AWMT, all essential oils induced faster paralysis of adult parasites than the negative controls [PBS + 4% antibiotic (penicillin-streptomycin) + 2% Tween 80; PBS + 4% antibiotic (penicillin-streptomycin)], completely inhibiting motility within the first 8–12 h of observation. In addition, a marked overall inhibition of motility of 50% or higher was observed for all oils within the first 2–4 h of the experiment (Table 4). The profile of anthelmintic activity of the oils was similar to that observed for the positive control, even when levamisole was used in a very high concentration (20 mg/ml) (Table 4).

**Table 4:** Percentage of motility of *H. contortus* adult worns when exposed to different concentrations of essential oils

		Time (hours)												
	Concentration (mg/ml)	0	2	4	6	8	10	12	14	16	18	20	22	24
Control (+)	–	100	49^b^	22^b^	9^b^	2^b^	0^b^	0^b^	0^b^	0^b^	0^b^	0^b^	0^b^	0
Control (-) 1	–	100^a^	100	91	84	71	53	40	31	22	16	11	7	0^a^
Control (-) 2	–	100^a^	100	91	80	64	51	33	24	18	13	4	2	0^a^
*C. aurantifolia*	50.0	100^a^	49^a,b^	27^a,b^	11^a,b^	7^a,b^	0^a,b^	0^a,b^	0^a,b^	0^a,b^	0^a,b^	0^a,b^	0^a,b^	0^a^
	5.0	100^a^	49^a,b^	27^a,b^	18^a,b^	9^a,b^	0^a,b^	0^a,b^	0^a,b^	0^a,b^	0^a,b^	0^a,b^	0^a,b^	0^a^
	0.5	100^a^	87^b^	58^b^	40^b^	13^b^	9^b^	0^a,b^	0^a,b^	0^a,b^	0^a,b^	0^a,b^	0^a,b^	0^a^
*A. nobilis*	50.0	100^a^	44^a,b^	13^a,b^	4^a,b^	0^a,b^	0^a,b^	0^a,b^	0^a,b^	0^a,b^	0^a,b^	0^a,b^	0^a,b^	0^a^
	5.0	100^a^	49^a,b^	18^a,b^	13^a,b^	0^a,b^	0^a,b^	0^a,b^	0^a,b^	0^a,b^	0^a,b^	0^a,b^	0^a,b^	0^a^
	0.5	100^a^	69^b^	38^b^	33^b^	16^b^	0^a,b^	0^a,b^	0^a,b^	0^a,b^	0^a,b^	0^a,b^	0^a,b^	0^a^
*L. officinalis*	50.0	100^a^	44^a,b^	31^a,b^	13^a,b^	7^a,b^	0^a,b^	0^a^	0^a,b^	0^a,b^	0^a,b^	0^a,b^	0^a,b^	0^a^
	5.0	100^a^	56^a,b^	36^b^	29^b^	1 ^a,b^	2^a,b^	0^a^	0^a,b^	0^a,b^	0^a,b^	0^a,b^	0^a,b^	0^a^
	0.5	100^a^	82^b^	54^b^	44^b^	24^b^	13^b^	9^a,b^	0^a,b^	0^a,b^	0^a,b^	0^a,b^	0^a,b^	0^a^

*Key*: Control (+), levamisole 20 mg/ml; Control (-) 1, PBS + antibiotic (penicillin/streptomycin) 4% + tween 80 2%; Control (-) 2, PBS + antibiotic (penicillin/streptomycin) 4%

^a^No statistically significant difference between positive control and treatments

^b^Significant difference between positive control or treatments with negative control (ANOVA, followed by Tukey test; *P <* 0.05)

## Discussion

The use of in vitro assays in veterinary parasitology, especially a series of tests for the evaluation of action against the main stages of a parasite (eggs, larvae and adults), is advantageous for the choice of substances to be tested in vivo [22]. These assays are rapid, inexpensive and simple. They reduce the number of animals that are necessary for in vivo testing, and permit the combined and optimized assessment of a series of compounds for both the development of new drugs and the study of resistance to different anthelmintics. Within this context, different approaches have used this experimental strategy for the study of anthelmintic agents, including those against *H. contortus* [23].

For the development of new anthelmintic agents, it is important that the parasite species, and even its strain, be the most representative of the livestock in reality, especially when this type of economical activity faces different challenges [4]. *Haemonchus contortus* strain, Embrapa 2010, is known to be resistant to the main classes of commercially available anthelmintic drugs, such as benzimidazoles, macrocyclic lactones and imidazothiazoles [17]. In fact, this anthelmintic resistance profile is very similar to that found on sheep farms throughout Brazil, particularly in the State of São Paulo [24].

Essential oils consist of a mixture of volatile and lipophilic compounds with a strong and characteristic odor. Terpenes and terpenoids are their main chemical constituents. These compounds differ by the presence of an oxygen atom in terpenoids and a methyl radical in terpenes [25]. In view of the low solubility of essential oils in water, a tensoactive solution or solvent was used to increase the solubilization of these substances in the present experiments. Tween 80 at 3% (v/v) and at 2% (v/v) was used in the EHT and AWMT, respectively, while 1% DMSO (v/v) was used in the LDT because L1 larvae are more sensitive to Tween 80 than to DMSO [19]. The negative and positive controls used in the different tests yielded the expected results, demonstrating appropriate standardization of the experimental protocol and, consequently, the reliability of the findings obtained (Tables 1, 2 and 3).

Human knowledge of essential oils originated in the pre-Christian era [10]. Anthelmintic properties, including against *H. contortus*, have been described for a large number of essential oils such as those of *Croton zehntneri* [26], *Eucalyptus staigeriana* [27], *Cymbopogon martinii* and *Mentha piperita* [28], *Lippia sidoides* [29] and *Thymus vulgaris* [30].

In the present study, the essential oil of *L. officinalis* was more effective in the EHT than the oils of *C. aurantifolia* and especially of *A. nobile*. In fact, the IC_50_ value of the *L. officinalis* oil was about 2.0–2.5 times lower than that of the other two oils tested (Table 2). In the LDT, the oil of *C. aurantifolia* exhibited the best performance, with an IC_50_ value that was about 2.5 and 6.5 times lower than those obtained for the oils of *A. nobile* and *L. officinalis*, respectively, and was therefore the most potent against larval development (Table 3). The variations in anthelmintic performance between the different tests and oils might be explained by qualitative and quantitative differences in the composition of the essential oils studied (Table 1) and different sensitivity to the various developmental stages of *H. contortus*. However, similar results were obtained in the AWMT for the oils tested, which did not permit a clear distinction of their pharmacological efficacy for this assay (Table 4). Furthermore, regardless of the variations in absolute IC_50_ values, all oils acted effectively and in a dose-dependent manner against the three developmental stages of *H. contortus*, which is a relevant finding.

Regarding the chemical compounds shown in Table 1, some of the volatile compounds identified are major constituents or are common in the different plant species studied. For example, limonene was identified as a major constituent in the oil of *C. aurantifolia* (56.37%). Limonene is characteristic of species of the genus *Citrus* [31]. However, although at low concentrations, limonene was also found in the oil of *L. officinalis* (0.52%) (Table 1). Myrcene was identified at low concentration in the oils of *C. aurantifolia* and *L. officinalis* (Table 1). Borneol, butyl acetate, camphene, 1,8-cineol and sabinene, also at low concentrations, can be found in both the oils of *A. nobilis* and of *L. officinalis* (Table 1). Except for α-thujene, which occurs in the oils of *C. aurantifolia*, *A. nobilis* and *L. officinalis* at proportions of 2.85%, 8.82% and 0.31%, respectively, no other compound identified occurred simultaneously in the three types of oils studied (Table 1), a finding supporting the chemical diversity of the oils. In fact, the essential oils studied here are composed of a wide diversity of compounds that represent a vast repertoire of active biomolecules of biotechnological interest. This permits the theoretical establishment of a natural product library for structure-activity correlation studies that are important for current pharmacology [32].

Linalool acetate and trans-sabinene hydrate are the major constituents of plants of the family Lamiaceae, especially in the genus *Lavandula* [33]. These were also the two main compounds identified in the oil of *L. officinalis* (35.97% and 29.17%, respectively) (Table 1). In addition to limonene (see above), other compounds present in the oil of *C. aurantifolia* should be mentioned, including γ-terpinene and β-pinene at proportions of 11.42% and 11.86%, respectively (Table 1). Two major constituents are present in the oil of *A. nobilis*, isobutyl angelate (29.26%) and isoamyl angelate (15.27%) (Table 1). These compounds, which are major constituents of all three oils studied, might be the main substances responsible for the different anthelmintic activities observed in this study. However, we cannot rule out that different compounds act synergistically to establish the final pharmacological effect in each case, or that compounds present in small or trace amounts are involved in the anthelmintic effect observed. In the latter case, α-thujene was not a major constituent in any of the oils studied, but was found in all three of them, although at variable low proportions (Table 1). This compound may represent a common functional link between the three different oils, all of them exhibiting anthelmintic activity.

In addition to the representative importance of major constituents of each oil studied, some of these compounds possess known anthelmintic properties, highlighting their potential importance in final anthelmintic activity. The evidence for the activity of such compounds can be direct or indirect, as observed for *H. contortus*. For example, limonene is found in the oils of different plants with anthelmintic activity, such as *Lippia sidoides*, *Cymbopogon martinii*, *Mentha piperita* and *Eucalyptus staigeriana* [19, 29, 34]. This compound accounts for 96.0% of the composition of the essential oil of *Citrus sinensis*, causing 100% inhibition of *H. contortus* eggs and larvae in vitro at most concentrations tested [35]. Linalool acetate is known for its anthelmintic activity against *Ascaris lumbricoides* [4]. Although not a major constituent of any of the oils, 1,8-cineole was present in both the oil of *A. nobilis* and *L. officinalis*. This compound is well known to be effective against the egg and larval stage of *H. contortus* [13].

In view of the economic impacts of gastrointestinal infections on sheep farming, which are increasingly aggravated by anthelmintic resistance, the identification and development of new therapeutic options for prevention, control or eradication of this problem is essential [10, 36]. In this respect, there has been intensified search for botanical anthelmintics against *H. contortus* because of the advantages of these agents, such as low cost and easy access, especially in less developed countries with biodiversity [37]. Since botanical anthelmintics may contain different compounds that act synergistically and throughout the life-cycle of the parasite, they may be less susceptible to pharmacological resistance, although this suggestion is controversial [22]. Finally, additional advantages are that botanical anthelmintics are generally well tolerated by animals from a toxicological point of view and appear to leave fewer residues in animal products such as meat and milk [37].

The application of ethnopharmacological/chemotaxonomic-based strategies is important to optimize studies of botanical anthelmintic agents in order to increase the success of developing new plant-derived or herbal agents of interest for the pharmaceutical-veterinary industry [10]. Although in vivo studies are still needed, the present work preliminarily validates the ethnopharmacological and chemotaxonomic data that indicate the use of *C. aurantifolia*, *A. nobile* and *L. officinalis* as anthelmintics. This is the first study testing the essential oils of these plants against *H. contortus*, highlighting their ethnopharmacological use and pharmaceutical-veterinary value.

## Conclusions

This study reveals the in vitro effects of *C. aurantifolia*, *A. nobile* and *L. officinais* essential oils against the main developmental stages of the parasite *H. contortus.* It highlights the importance of promising medicinal plants and theirs chemical compounds for the development of new potential anthelmintic agents.

## Abbreviations

AWMI: Adul worm motility test
DMSO: Dimethyl sulfoxide
EHT: Egg hatch test
EPG: Eggs per gram of feces
GC-MS: Gas chromatography coupled to mass spectrometry
LDT: Larval development test
PBS: Phosphate-buffered solution
